# Analyzing longitudinal qualitative data: the application of trajectory and recurrent cross-sectional approaches

**DOI:** 10.1186/s13104-016-1954-1

**Published:** 2016-03-02

**Authors:** Daniel Grossoehme, Ellen Lipstein

**Affiliations:** Department of Pediatrics, University of Cincinnati College of Medicine, Cincinnati, OH USA; Division of Pulmonary Medicine, Cincinnati Children’s Hospital Medical Center, MLC2021, 3333 Burnet Avenue, Cincinnati, OH 45229 USA; James M. Anderson Center for Health Systems Excellence, Cincinnati Children’s Hospital Medical Center, 3333 Burnet Avenue, Cincinnati, OH 45229 USA; Division of Adolescent and Transition Medicine, Center for Innovation in Chronic Disease Care, Cincinnati Children’s Hospital Medical Center, 3333 Burnet Avenue, Cincinnati, OH 45229 USA

**Keywords:** Longitudinal, Qualitative methodology, Health services research

## Abstract

**Background:**

Longitudinal qualitative research methods can add depth and understanding to health care research, especially on topics such as chronic conditions, adherence and changing health policies. In this manuscript we describe when and how to undertake two different applied approaches to analyzing longitudinal qualitative data: a recurrent cross-sectional approach and a trajectory approach.

**Results:**

A recurrent cross-sectional approach is most appropriate when the primary interest is comparing two time points, such as before and after a policy change, or when a cohort cannot be maintained, such as a study in which some participants are expected to die. In contrast, a trajectory approach is most appropriate when the purpose of the research is to understand individuals’ experiences over time or to understand longitudinal healthcare processes.

**Conclusions:**

Longitudinal qualitative research has the potential to be a powerful approach to understanding the complexities of health care: from relationships between providers and patients, to the experience of chronic disease, to the impact of health policy. Such research will be strengthened by careful consideration of the research question at hand, followed by application of the appropriate analytic approach.

## Findings

### Background

Qualitative research is an essential part of applied health care research [1, 2]. The in-depth approaches used in qualitative research allow for a better understanding of the lived experience of disease, including the ways in which individuals interacted with the health care system and why they made specific health care choices. This research then helps generate hypotheses for future study and ultimately leads to improvements in health and health systems.

Within health care, most qualitative studies are cross-sectional. They employ a variety of data collection methods, such as interviews or focus groups, and the analyses often focus on understanding experiences in a specific time and place, or on participants’ recollections of prior experiences. However, typically, individuals’ experiences with health and the health care system occur over time. Therefore, a prospective understanding of the longitudinal experience may provide insight and direction that differs from that of cross-sectional data. For example, researchers have used longitudinal qualitative methods to examine decision making [3] and information needs [4] in cancer care, medication adherence, [5] and the health care experiences children with serious health conditions [6, 7].

Existing studies in the healthcare literature often have sparse methods descriptions, making it hard for others to replicate a study or emulate the methods in a new study. Additionally, many citations for analytic methods come from books and studies where the applicability to health care research may not obvious. For these reasons, we sought to describe and clarify applied qualitative research methods that are accessible to researchers focused on health service research, medicine and health, or health care. In this manuscript we provide detailed explanation of two different approaches to longitudinal qualitative data analysis: a *recurrent cross*-*sectional approach,* for analyzing group-level data, and a *trajectory approach,* focused on individuals’ or small groups’ of individuals (e.g., families) experiences over time. By providing straightforward explanations of these methods, we hope to assist other researchers interested in understanding changes in health and healthcare experiences over time, through pursuit of longitudinal qualitative research.

### Choosing an analytic approach

For the purposes of this article, we assumed that there are two primary approaches to analyzing longitudinal qualitative data: recurrent cross-sectional and trajectory (see Table 1). Although likely to be less common, there may be some specific research questions that are amenable to the researcher combining the two approaches. As in any research analysis, the key to determining the right approach is considering the focus of the research question.

**Table 1:** Comparison of recurrent cross-sectional and trajectory analysis

Considerations	Recurrent cross-sectional analysis	Trajectory analysis
Research focus	Describe the differences between time points	Describe how process or experience changes over time
Sample considerations	The cohort at each time point may be the same or different May be preferred if sample is highly transient or has high mortality over study duration	Must maintain same cohort
Theoretical approach	Determined by the research question Any analytic approach may be used consistently throughout the study	Determined by the research question Any analytic approach may be used consistently throughout the study
Level of data analysis	Whole sample (or subsamples)	Individual people or individual groups (e.g., families)
Timing of analysis	May analyze as each time point is completed	Must wait until data is collected at all time points

*Recurrent cross*-*sectional analysis* explores themes and changes over time at the level of the entire study sample, although there may also be variation of interest in the samples at different time points. If the researcher’s primary interest is comparing two time points then cross-sectional analysis is likely preferred. For example, research seeking to understand reactions to a new health guideline might want to include a cross-sectional analysis from before and after implementation of the guideline. Additionally, questions of how group-level beliefs change over time, for example how “healthy eating” is defined, might be addressed appropriately in a recurrent cross-sectional study. Finally, there are situations in which maintaining a cohort is not feasible, either because of a long time span or because of the subject of the study. The latter is exemplified in a study by Ragsdale and colleagues of children under-going bone marrow transplant [8]. The authors knew that participants might die prior to follow-up and thus a cross-sectional design was more feasible, in order to not exclude participants who were interviewed early in the study and subsequently died.

*Trajectory analysis* focuses on changes over time for an individual or small group of individuals. When the purpose of the research is to understand individuals’ experiences over time or to understand longitudinal healthcare processes, we recommend using a trajectory approach. When the research interest is an experience or process, and the reactions to it, it becomes important to conduct analyses in a manner that emphasizes individual trajectories. For example, one of the authors (EL) has conducted research focused on decision making in chronic conditions [9]. By following the individual trajectories, we found that the factors influencing individuals’ decisions vary over time. Had we instead analyzed the data as recurrent cross-sections, such change would not have been visible because the factors considered by the population as a whole did not change; rather individuals changed the factor on which they focused. Likewise, if a study were designed to understand how individuals’ cope with test results, such as from cancer screening tests, cross-sectional analysis could lead to erroneous conclusions by focusing on how individuals are doing at a set time after testing, rather than on their experience over time. While some shifts in preferences or attitudes could be ascertained through individuals’ comments about the past, such recall is subject to significant bias [10]. Moreover, individuals are often unaware of how their perspective has changed over time based on experiences and information [11, 12].

### Setting up the analysis: data coding

Regardless of the chosen analytic approach, cross-sectional or trajectory, prior to beginning data collection the research team should consider both their research question, as discussed above, and the theoretical approach they plan to use for analyzing the data (e.g., grounded theory. This a priori decision making will ensure that data is collected, coded and structured in a manner consistent with the research plan. This coding step is amenable to an array of theoretical (e.g., grounded theory or phenomenological) and practical (e.g., using software or coding by hand) approaches.

For either analytic approach, utilizing a framework [13] or a list of analytic questions [14] may assist in structuring the data. For example, Saldaña’s often-cited reference for longitudinal qualitative research outlines 16 questions to help structure the analytic process. These questions can be divided into framing questions, descriptive questions and interpretive questions, all of which can be applied to either method of analyzing longitudinal data. Framing questions situate the context of the data and the health care process in which the data have arisen. Typical framing questions include describing how data collected at each time point relates to data from the other time points (e.g., defining changes in context, or when changes occur). Descriptive questions are intended to guide the interpretive phase of data analysis. The answers to these questions describe behavior in a particular environment [15]. Interpretive questions lead to descriptions of the behavior of interest within its context of relationships. These may include how changes in the behavior relate to one another; mediators and barriers to the behavior; or the data’s consistency with current practices.

### Conducting a recurrent cross-sectional analysis

The recurrent cross-sectional approach has been the more commonly used approach to longitudinal qualitative research in healthcare. The analytic process is very similar to studies that focus on a single point in time, so details of this approach will only briefly be discussed. For readers interested in more in-depth guidance on cross-sectional approaches many good resources have been published including qualitative research and evaluation methods by Patton [16], qualitative data analysis by Miles and Huberman [17], and constructing grounded theory by Charmaz [18]. The recurrent cross-sectional approach can be thought of as a series of smaller studies study given that at each time point the data from all participants are analyzed as a unit. After this analysis is completed, a second analysis focuses on differences and similarities between time points [14]. A potential advantage of this approach is that analysis of early time points can be completed before data is even collected for subsequent time points. For example, one of the authors (DG) conducted a study of how parents of children used spirituality to cope in the first 12 months after their child’s cystic fibrosis diagnosis. We found four major themes related to, “We can handle this” [19]. After an additional 12 months elapsed, parents were re-interviewed; the longitudinal analysis showed parents now understood themselves to have been “chosen” to parent a child with CF [20]. Both the initial study and the follow-up study were independent grounded theory analyses of parental interview data [18]. In each case, transcripts were coded line by line by a team to isolate participants’ descriptions in their own words. These fragments were grouped into categories based on apparent similarity. These categories were further combined and a central theme that explained the emergent major categories was identified. The follow-up study also included a second analysis using Saldaña’s framing, descriptive, and interpretive questions. For example, the central themes of the two studies (“We can handle this” and “We were chosen as a family”) were placed next to each other and the framing question of differences between the time points explored. While one might have anticipated increasing confidence in parents’ coping and caring for a child with a life-shortening disease, what this method made clear was that their confidence was expressed in terms of religious vocation. Applying Saldaña’s questions to both sets of data allowed for the creation of a richer, single narrative that focused on the process of coping spanning 2 years.

### Conducting a trajectory analysis

To our knowledge, few studies have utilized a trajectory approach despite the importance of individuals’ longitudinal experiences in the healthcare system. Moreover, the limited methods descriptions in many such studies may make it difficult for other researchers, especially those new to qualitative research, to reproduce the methods in their own work. For these reasons, we have developed a more structured approach to trajectory analysis that could be utilized by those new to the field. Specifically, we recommend using time-ordered, sequential matrices. Time-ordered displays have been previously described as a method to help preserve “chronological flow” and permit understanding of what led to what [17]. Trajectory analysis expands upon this base through the use of sequential matrices.

Once coded the data is organized into matrices, with one matrix per unit of analysis. The unit of analysis could be the individual, the family, or some other grouping of people. A combination could also be considered. For instance, one may be interested in how similar or different the experience is for individuals within a family. In that case, each matrix should contain the largest unit of analysis (example family) and codes from individuals within that group which can be identified via labeling. The use of color coding or font variations is recommended, rather than text labels, in order to facilitate a visual overview of the data. However, some software programs used for data analysis may not permit such visual labeling. This first set of matrices is organized with themes, based on your theoretical approach, along the Y-axis and time along the X-axis (see Table 2). To illustrate the basic principle of the matrix, themes which occurred at all three points are presented in Table 1, although it may be helpful for some research questions to include topics which occur at only some time points.

**Table 2:** Sample family matrix

Themes	Time 1	Time 2	Time 3
Theme A (example: family stress)	Lots of stress about health	Feeling stressed about treatment decision *Feeling stressed about treatment decision*	*Less stressed now that decision is made*
Theme B (example: concerns about side effects)	*Worried how treatment will impact growth*	No concerns about side effects *Concerned about child’s growth*	No concerns about side effects *Less worried about side effects since child is improving*
Theme C	Idea from mother *Idea from father*	*Idea from father*	Idea from mother
Theme D	Idea from mother *Idea from father*	*Idea from father*	Idea from mother *Idea from father*

Plain font indicates mother; *italics* indicates father

Once data collection and coding are completed for each unit of analysis, longitudinal analysis can begin. In this step the focus is on how the data, in the thematic groupings, changed or did not change over time. To organize the findings, another matrix is needed. The Y-axis is again organized by themes. This time the X-axis is organized according to the primary units of analysis, in other words one column per unit of analysis (see Table 3). If the first set of matrices included labels for individuals within a unit of analysis, that labeling should continue in this matrix. The data codes entered in this matrix focus on the element of time. Codes may be used that indicate concepts that change over time or remain stable. For instance if a theme in the first set of matrices was family stress, the codes in the second matrix would focus on increases or decreases in stress over time. In this step it is particularly important to pay attention to data absences in the first set of matrices [14]. This may not indicate a deficit in coding but rather signal variation over time. As an example, if a participant discusses concerns about side effects of treatment at time one, but not at time two, it may indicate, depending upon how the data was collected, that the concern about side effects has dissipated over time.

**Table 3:** Sample longitudinal analysis matrix

Themes	Family 1	Family 2	Family 3
Theme A (example: change in family stress over time)	Change from stress about health to stress about treatment *Moved towards less stress after treatment started*	Idea from mother *Idea from father*	Idea from mother
Theme B (example: change in concerns about side effects over time)	Never developed any concerns *Worried about growth that diminishes as child improves*	Idea from mother *Idea from father*	Idea from mother
Theme C	Idea from mother	Idea from mother *Idea from father*	Idea from mother
Theme D	Idea from mother *Idea from father*	Idea from mother *Idea from father*	Idea from mother

Plain font indicates mother; *italics* indicates father

As in most qualitative approaches, as coding for the second matrix progresses, new conceptual groupings may be needed as the original groupings likely focused on cross-sectional concepts and new, time-related concepts may emerge during coding. Data analysis is then conducted from this second matrix in which the codes are focused on time, with reference back to the first set of matrices when specific examples are needed.

### Conclusions

Longitudinal qualitative research has the potential to be a powerful approach to understanding the complexities of health care: from relationships between providers and patients, to the experience of chronic disease, to the impact of health policy. This research will be strengthened by careful consideration of the research question at hand, followed by application of the appropriate analytic approach. A recurrent cross-sectional approach is best utilized for questions that focus on comparing discrete time points or where logistical challenges prevent retention of a research cohort. However, when the focus is on how experiences or processes unfold over time, a trajectory approach should be considered. A lack of methodological clarity in published studies has been a barrier to undertaking such research and potentially limited its impact. By presenting the rationale for using longitudinal qualitative methods, their description, accompanying examples and citations we hope to stimulate use of these methods to further enhance health care research.
